# Referral compliance and subsequent hospital admissions for COPD and cardiovascular disease in the Netherlands: a data linkage study

**DOI:** 10.1186/s12913-025-13391-4

**Published:** 2025-10-09

**Authors:** J. T. Dros, C. E. van Dijk, R. A. Verheij, I. Bos, B. R. Meijboom

**Affiliations:** 1 Netherlands Institute for Health Services Research (NIVEL), Utrecht, Netherlands; 2 National Health Care Institute, Diemen, Netherlands; 3 Tilburg School of Social and Behavioral Sciences, Tranzo, Tilburg University, Tilburg, Netherlands

**Keywords:** Cardiovascular diseases, Chronic obstructive pulmonary disease, Specialty referrals, Referral compliance, Hospital admissions

## Abstract

**Background:**

General practitioners act as gatekeepers in the Dutch healthcare system, ensuring timely access to specialist care. Few studies have been conducted on compliance with specialty referrals, particularly for conditions like chronic obstructive pulmonary disease (COPD) and cardiovascular disease (CVD), where timely specialist intervention is often important. Therefore, the aim of this study was to gain deeper insight into specialty referral compliance and associated factors, as well as the association between time to compliance and specialist care utilization for COPD and CVD.

**Methods:**

We linked medical claims data from Dutch health insurers and electronic health records from general practice over the period 2015–2020. To analyze specialty referral compliance and healthcare utilization patterns for patients with COPD or at high risk of CVD, referral compliance and healthcare use were assessed over a two-year follow-up period. We adjusted for case-mix factors and time in the dataset.

**Results:**

Among COPD patients, 1113 of the 35,606 patients (3.1%) were referred to medical specialist care. Thereof, 5.2% did not comply within one year. Patients that had not depleted their deductibles were more likely to either comply later or not comply altogether. Time to compliance was not associated with hospital admissions, but when compliance was delayed between 1 and 3 months, Intensive Care Unit (ICU) admissions did become more likely. Among patients at high risk of CVD, 2194 of the 130,540 patients (1.7%) were referred to specialist care, with 8.8% remaining non-compliant after one year. Surprisingly, depletion of deductibles increased the likelihood of non-compliance. For both COPD and CVD, age and comorbidities had significant but varying associations with referral compliance.

**Conclusion:**

Our findings suggest that age, socioeconomic status, financial barriers or certain comorbidities can delay or prohibit compliance, with potential adverse outcomes like ICU admissions. Future research should explore whether non-compliant patients experience worse health outcomes beyond two years or if their conditions are managed without specialist care.

**Supplementary Information:**

The online version contains supplementary material available at 10.1186/s12913-025-13391-4.

## Background

In many healthcare systems around the world, and especially in the Netherlands, general practitioners (GPs) function as gatekeepers [1]. They assess whether specialist care is warranted based on their broad clinical expertise. When specialist care is needed, patients are referred for further diagnostics treatment and care. Prompt compliance to specialty referrals may prevent further progression of diseases [2]. Patient behavior in response to referrals directly influences the use for services. When diseases are not treated, this might potentially lead to more severe outcomes and higher healthcare costs. Therefore, prompt compliance does not only benefit patients, but it is also essential for a sustainable healthcare system. Thus, gaining insight into this behavior, the associated factors, and the influence on consequent healthcare use is relevant for resource allocation.

Previous studies show large practice variation in GPs’ referral rates to medical specialists [2–4]. Only few studies report on specialty referral compliance. Forrest et al. (2007), found that around 17–21% of patients do not follow-up on their specialty referral in the US [2]. In 2017, van Dijk et al. reported a somewhat higher compliance rate in the Netherlands, with only 13% of specialty referrals not being complied to between 2008 and 2010^5^. These differences in compliance rate might be related to the differences in patient populations and healthcare systems. Financial policies such as out-of-pocket expenses or deductibles may also play an important role. In the Dutch health care system, basic health insurance is mandatory. A deductible is in place for the first €385 (in 2019), except for GP consultations and GP out of hours services, maternity care, home nursing care, and care for children under the age of 18. This means that patients referred early in the calendar year may face more out-of-pocket expenses, potentially leading to delayed or foregone care.

An earlier Dutch study by Van Esch et al. (2017), suggests that high deductibles might discourage individuals from complying with specialty referral [5]. Van Esch et al. report the number of patients complying with referrals and not the number of referrals complied with, which was done by van Dijk et al. They found that noncompliance increased after a rise of the compulsory deductible that had to be paid in the Netherlands which went from 18 to 26% between 2010 and 2012. The compulsory deductible has stabilized after the study by van Esch et al. More recent numbers on referral compliance are lacking.

Referral rates reported in earlier studies are difficult to compare, since they do not only differ in level of analyses (i.e. patient or referral level), but also in the chosen timeframe for compliance. Where Forrest et al. determined compliance three months after referral, van Dijk et al. and van Esch et al. determined compliance using a six month period after referral. However, patients may initially delay their follow-up due to a variety of reasons like lack of time, the belief that the health problem has resolved or long waiting times [2, 6]. The duration of this delay can vary, suggesting that it might be interesting to study compliance over a longer period. The chosen timeframe in the Netherlands is even more important, since the compulsory deductible has to be paid annually and resets each year. This creates a financial incentive for patients to delay compliance for non-urgent referrals until later in the calendar year when their deductibles might already be depleted. Or they might wait until the next year, if they know they will deplete their deductibles that year anyway due to ‘planned’ procedures. Hence, they might comply later than six months after their referral. Additionally, such incentives might influence health seeking behavior different throughout the year, which was also posed by van Esch et al. [5]. Therefore, allowing for a compliance period of one year or more might allow to incorporate these dynamics better.

Compliance with specialty referral can play a crucial role in intervention for conditions requiring timely care, like for instance chronic obstructive disease (COPD), in which acute exacerbations accelerate disease severity [7]. In medical specialist care, patients with COPD receive medication and pulmonary rehabilitation, which can improve the quality of life, reduce the risk for readmission after a recent exacerbation and shows a strong reduction in mood- and anxiety symptoms [8–10]. Prompt compliance is also important for patients at high risk of cardiovascular disease (CVD), although the care utilization patterns of these patients differ substantially from patients with COPD. Timely access to specialized medical care can mitigate disease progression and reduce adverse outcomes like a cardiovascular event [11]. Due to their high prevalence and chronicity these conditions are well-suited to explore specialty referral compliance.

In the Netherlands, studies on specialty referral compliance for COPD and CVD and consequences associated with noncompliance are unknown. Therefore, the aims of this study are: (1) to gain insight into which patients with COPD and at high risk of cardiovascular diseases are referred to medical specialist care, (2) to gain deeper insight into specialty referral compliance and associated factors for these patients, and (3) to gain insight into the association between time to specialty referral compliance and hospital admissions. We will analyze referral rates from general practice to medical specialist care, along with the time elapsed between referral and specialist consultation. We will explore referral compliance, defined as the time elapsed between referral and medical specialist consultation. Also, we will provide insight into the healthcare utilization patterns that are associated with time to referral compliance. To our knowledge, we are the first to study referral compliance at a disease specific level.

## Methods

### Study design

In this observational study, we linked individual patient’s medical claims data from medical specialists and general practices to electronic health records (EHRs) from general practice. The data linkage process is described in our previous paper (Dros et al., 2024). Vektis (the center for information of Dutch Health insurers, which collects data including insurance claims of general practices and medical specialists) provided the medical claims data through the National Healthcare Institute (NHCI, in Dutch: Zorginstituut Nederland). The Nivel Primary Care Database (Nivel-PCD) provided the EHR data, consisting of patient data from a representative sample of 10% of the Dutch population [12]. Referral information is only included in electronic patient records of ‘Zorgdomein’ (a software system for referring patients) practices, which is available for roughly 25% of all general practices in the Nivel-PCD. Hence, the analyses in this paper are conducted on a subset of 2.5% of the Dutch population. Patients in Zorgdomein practices have slightly higher neighborhood socioeconomic status (SES) and age, for which we adjust in our analyses as described in the *variables* section below.

### Patient sample

We studied patients with a referral between 2015 and 2018. Patients were followed for two years after their initial referral date. Hence, the study period was 2015–2020. We selected patients with existing CVD and COPD from EHRs and medical claims, meaning that patients with multiple years of referral can be included in the dataset for several years. We focused on existing COPD patients and on CVD patients at high risk of experiencing a cardiovascular event, but who had not yet undergone an event yet. Patient were classified as having COPD if they met the following criteria:

- They were diagnosed with an International Classification of Primary Care (ICPC) code or Diagnosis-Related Group (DRG) code for COPD, and;
- They were at least 40 years old. For younger patients, asthma or related conditions are expected to frequently interfere with diagnostic registration, increasing the risk for misclassification.

Patients were classified as high risk if they met the following criteria:

- They had not experienced a cardiovascular event, as defined in our earlier paper (Dros et al., 2024)^1^, and;
- They did not receive kidney dialysis or a kidney- or heart transplantation, and;
- They were at least 18 years old, and;
- They were diagnosed with at least one cardiovascular risk factor (based on DRG codes), or;
- They were diagnosed with hypertension (ICPC code or DRG) and had a prescription for hypertension (Anatomical Therapeutic Chemical (ATC) codes C02, C03, C07, C08, and/or C09), or;
- They were diagnosed with hypercholesterolemia (ICPC code or DRG) and had a prescription for hypercholesterolemia (ATC code C10).

This classification is based on the ICPC codes, ATC codes, and DRGs listed in Additional File I.

### Variables

We measured the following outcomes: (1) specialty referral rate; (2) referral compliance rates per compliance period; (3) specialty healthcare use for patients within 24 months after referral per compliance periods. The referral compliance was determined by using the referral date recorded in the GP EHR data, and the actual usage of medical specialist care for which patients were referred in claims data (see Additional File II for referral and medical specialist codes). In contrast to earlier studies, we did not set a definitive compliance period. Rather, we set several compliance periods. In order to compare our results with previous studies, we calculated referral compliance within 3 and 6 months [2, 5, 13]. However, in the histogram in Additional File III, it can be seen that many patients comply immediately in the day of referral, and also that many patients comply within one month. Also, some patients still comply after more than 6 months. Hence, the following compliance periods were chosen: within 1 day, between 1 day and 1 month, between 1 and 3 months, between 3 and 6 months, and between 6 and 12 months. Healthcare use after referral was determined as the type of hospital consultations for patients within two years after their referral date. These consultations were based on ‘care activities’ from claims data, from which we obtained the following consultations: diagnostic, hospital admission, intensive care unit (ICU) admission, surgery or day treatment. As adverse events we tested hospital and ICU admissions as a function of time to compliance, which we describe in the statistical analyses section.

In order to gain more insight into factors associated with specialty referral compliance, we also included case-mix variables in the analyses. We included age, gender, neighborhood SES, calendar year, depletion of deductibles, distance to nearest hospital and comorbidities. Referrals and comorbidities were derived from Nivel-PCD EHR data. Comorbidities were included in the year of referral and their relevance was based on medical guidelines [14–20]which differed for COPD and CVD. Medical specialist care consultations (‘care activities’), depletion of deductibles, age, gender and distance to nearest hospital were derived from claims data. Depletion of deductibles was determined by subtracting GP consultations, maternity care and home nursing care costs from the total annual healthcare costs, since patients are exempted to pay deductibles for care from these providers. Costs attributed to medical specialist care specific for respectively CVD or COPD were also subtracted from total annual healthcare costs. The neighborhood SES score was also included, as a relative number of the standing of a neighborhood relative to other neighborhoods and was determined by education, income, and employment status. These scores are categorized into quartiles, with a higher quartile denoting a higher SES level [21]. The number of years that patients are included in the dataset varies due to death, treatment in nursing homes or incomplete claims or EHR data from insurers or general practices respectively. Therefore, all analyses were corrected for time in the dataset (see Additional File IV).

### Statistical analyses

We conducted descriptive analyses for all outcomes. We ran multinominal logistic regression models for case-mix factors associated with referral compliance time. Patients that were in need of acute care (defined as complying immediately on the referral date) were excluded from these analyses. Case-mix variables were age, gender, neighborhood socioeconomic status (SES), comorbidities and distance from patients to the nearest hospital. For specialty healthcare use within 24 months after referral, we ran multilevel logistic regression models with the compliance period as main determinant, adjusted for case-mix. This regression was only ran for compliant patients, since non-compliant patients do not utilize hospital care by definition. We added a dichotomous variable to our regression models indicating whether a patient’s compliance period overlapped with the first COVID-19 wave in the Netherlands (coded as 1 = during the wave, 0 = outside the wave). This allowed us to account for possible effects of delayed or disrupted care related to the pandemic. Analyses were performed using SAS Enterprise version 8.3 and R 4.1.2 (RStudio Pro, version 2023.12.1).

## Results

### Patients with chronic obstructive pulmonary disease

The study population for COPD consisted of 35,606 patient years between 2015 and 2018 (Table 1). Patients were evenly divided between the age groups 40–64, 65–74 and 75 + years old, with slightly more males. Neighborhood socioeconomic status (SES) was relatively low, with most patients in the low (35.3%) and medium-low (28.8%) group.

**Table 1:** Characteristics of prevalent patients with chronic obstructive pulmonary disease referred between 2015–2018, by referral status

	Non-referred (*N* = 34,493)	Referred (*N* = 1113)	Overall (*N* = 35,606)
Age in years (categorical)			
40–64	11,579 (33.6%)	405 (36.4%)	11,984 (33.7%)
65–74	11,294 (32.7%)	394 (35.4%)	11,688 (32.8%)
75+	11,620 (33.7%)	314 (28.2%)	11,934 (33.5%)
Female	16,797 (48.7%)	557 (50.0%)	17,354 (48.7%)
Socioeconomic status (SES)			
Low	12,192 (35.3%)	441 (39.6%)	12,633 (35.5%)
Medium-low	9921 (28.8%)	327 (29.4%)	10,248 (28.8%)
Medium-high	7441 (21.6%)	215 (19.3%)	7656 (21.5%)
High	4939 (14.3%)	130 (11.7%)	5069 (14.2%)
Deductibles depleted	30,837 (89.4%)	1093 (98.2%)	31,930 (89.7%)
Comorbidities for COPD			
Mood or anxiety	6398 (18.5%)	234 (21.0%)	6632 (18.6%)
Asthma	8258 (23.9%)	243 (21.8%)	8501 (23.9%)
Lung carcinoma	1319 (3.8%)	59 (5.3%)	1378 (3.9%)
Obesity	847 (2.5%)	20 (1.8%)	867 (2.4%)
Cardiovascular risk factors	22,691 (65.8%)	686 (61.6%)	23,377 (65.7%)
Diabetes mellitus type II	7593 (22.0%)	219 (19.7%)	7812 (21.9%)
Osteoporosis	3996 (11.6%)	160 (14.4%)	4156 (11.7%)

### Referrals

Between 2015 and 2018, a total of 1,113 (3.1%) patients were referred and 34,493 (96.9%) were not referred (Table 1). Referred and non-referred patients mostly differed in age, where referred patients were more often between 40 and 74 than non-referred patients. Patients who lived in neighborhoods with low socioeconomic status formed a substantial proportion of our study population and constitute a large proportion of the referred patients. A substantially (98.2% vs. 89.4%) higher percentage of referred patients had depleted their deductibles in the year of referral (excluding the medical specialist care for which they were referred – see method section). The most common comorbidities in both groups were cardiovascular risk factors, asthma and diabetes type II, which were all more prevalent among non-referred patients. Mood- and anxiety disorders, lung carcinoma and osteoporosis were more prevalent among referred patients.

### Referral compliance and associated factors

The days between referral date and start of the hospital treatment episode for compliant patients are shown in Additional File III. In 13.7% of the referrals, patients complied immediately on the day of referral, suggesting the need for acute care (Table 2). Between 1 and 30 days after referral, the compliance rate was 47.5%. After 3 months, most patients had complied with their referral (86.8%). Few more patients complied between 3 and 12 months (8.0%). Ultimately, 5.2% of patients did not comply with their referral within 1 year.

**Table 2:** Characteristics and healthcare use of patients with chronic obstructive pulmonary disease referred between 2015–2018

	Immediate	Between 1–30 days	Between 1–3 months	Between 3–6 months	Between 6–12 months	> 1 year or non-compliant		Overall
Number of patients (compliance rate)	152 (13.7%)	529 (47.5%)	285 (25.6%)	46 (4.1%)	43 (3.9%)	58 (5.2%)		1113
Age in years (categorical)								
40–64	38 (25.0%)	203 (38.4%)	112 (39.3%)	16 (34.8%)	12 (27.9%)		24 (41.4%)	405 (36.4%)
65–74	66 (43.4%)	173 (32.7%)	101 (35.4%)	20 (43.5%)	22 (51.2%)*+		12 (20.7%)	394 (35.4%)
75+	48 (31.6%)	153 (28.9%)	72 (25.3%)	10 (21.7%)	9 (20.9%)		22 (37.9%)	314 (28.2%)
Female	82 (53.9%)	251 (47.4%)	142 (49.8%)	22 (47.8%)	24 (55.8%)		36 (62.1%)	557 (50.0%)
Socioeconomic status (SES)								
Low	59 (38.8%)	210 (39.7%)	112 (39.3%)	15 (32.6%)	19 (44.2%)		26 (44.8%)	441 (39.6%)
Medium-low	48 (31.6%)	154 (29.1%)	83 (29.1%)	13 (28.3%)	12 (27.9%)		17 (29.3%)	327 (29.4%)
Medium-high	32 (21.1%)	103 (19.5%)	50 (17.5%)	13 (28.3%)	8 (18.6%)		9 (15.5%)	215 (19.3%)
High	13 (8.6%)	62 (11.7%)	40 (14.0%)	5 (10.9%)	4 (9.3%)		6 (10.3%)	130 (11.7%)
Deductibles depleted	151 (99.3%)	525 (99.2%)	275 (96.5%)*-	45 (97.8%)	42 (97.7%)		55 (94.8%)*-	1093 (98.2%)
Quarter of referral								
Q1 (January – March)	55 (36.2%)	158 (29.9%)	65 (22.8%)	21 (45.7%)	13 (30.2%)		14 (24.1%)	326 (29.3%)
Q2 (April – June)	33 (21.7%)	141 (26.7%)	91 (31.9%)*+	9 (19.6%)*-	14 (32.6%)		18 (31.0%)	306 (27.5%)
Q3 (July – September	21 (13.8%)	115 (21.7%)	72 (25.3%)	3 (6.5%)*-	8 (18.6%)		11 (19.0%)	230 (20.7%)
Q4 (October – December)	43 (28.3%)	115 (21.7%)	57 (20.0%)	13 (28.3%)	8 (18.6%)		15 (25.9%)	251 (22.6%)
Distance to nearest hospital in kilometres	9.8 (7.6)	9.9 (8.0)	10.2 (8.0)	8.5 (5.7)	8.8 (8.4)		8.5 (7.3)	9.8 (7.8)
Comorbidities								
Mood or anxiety	37 (24.3%)	106 (20.0%)	55 (19.3%)	7 (15.2%)	10 (23.3%)		19 (32.8%)	234 (21.0%)
Asthma	24 (15.8%)	116 (21.9%)	66 (23.2%)	12 (26.1%)	12 (27.9%)		13 (22.4%)	243 (21.8%)
Lung carcinoma	15 (9.9%)	31 (5.9%)	9 (3.2%)	2 (4.3%)	1 (2.3%)		1 (1.7%)	59 (5.3%)
Cardiovascular risk factors	96 (63.2%)	344 (65.0%)	160 (56.1%)*-	26 (56.5%)	27 (62.8%)		33 (56.9%)	686 (61.6%)
Diabetes mellitus type II	38 (25.0%)	105 (19.8%)	48 (16.8%)	5 (10.9%)	10 (23.3%)		13 (22.4%)	219 (19.7%)
Osteoporosis	26 (17.1%)	67 (12.7%)	39 (13.7%)	9 (19.6%)	6 (14.0%)		13 (22.4%)	160 (14.4%)
Specialist consultations < 2 year after referral								
Diagnostics	150 (98.7%)	517 (97.7%)	280 (98.2%)	44 (95.7%)	40 (93.0%)		13 (22.4%)	1044 (93.8%)
Admission	123 (80.9%)	171 (32.3%)	102 (35.8%)	15 (32.6%)	16 (37.2%)		5 (8.6%)	432 (38.8%)
* Short (0–5 days)*	27 (17.8%)	47 (8.9%)	24 (8.4%)	6 (13.0%)	5 (11.6%)		3 (5.2%)	112 (10.1%)
* Moderate (5–28 days)*	77 (50.7%)	102 (19.3%)	61 (21.4%)	7 (15.2%)	11 (25.6%)		2 (3.4%)	260 (23.4%)
* Long (> 28 days)*	19 (12.5%)	22 (4.2%)	17 (6.0%)	2 (4.3%)	0 (0%)		0 (0%)	60 (5.4%)
ICU admission	15 (9.9%)	14 (2.6%)	17 (6.0%)	1 (2.2%)	1 (2.3%)		0 (0%)	48 (4.3%)
* Short (0–5 days)*	10 (6.6%)	11 (2.1%)	8 (2.8%)	1 (2.2%)	1 (2.3%)		0 (0%)	31 (2.8%)
* Moderate (5–28 days)*	4 (2.6%)	3 (0.6%)	8 (2.8%)	0 (0%)	0 (0%)		0 (0%)	15 (1.3%)
* Long (> 28 days)*	1 (0.7%)	0 (0%)	1 (0.4%)	0 (0%)	0 (0%)		0 (0%)	2 (0.2%)
Surgery	2 (1.3%)	7 (1.3%)	4 (1.4%)	0 (0%)	2 (4.7%)		1 (1.7%)	16 (1.4%)
Day treatment	8 (5.3%)	34 (6.4%)	17 (6.0%)	1 (2.2%)	2 (4.7%)		1 (1.7%)	63 (5.7%)

*negatively (-) or positively (+) associated with complying in the respective referral period (ref: 1–30 days), based on the multinominal regression analyses as shown in Additional File V

Multinominal regression analyses (Additional File V, Table A5-1) show that several factors were significantly associated with time to referral compliance, as compared to compliance between 1 and 30 days. Patients that immediately complied on the referral date were excluded from these analyses, as they were most likely in need of acute care. This is confirmed by the hospital and ICU admissions rates within this group. Hence, they likely differed substantially from patients that did not immediately comply, which is also suggested by the admission rates falling substantially in these groups. The reference category therefore was referral compliance between 1 and 30 days. Patients that had depleted their deductibles (OR 0.22 [0.07–0.72]) and patients that had cardiovascular risk factors (OR 0.70 [0.51–0.96]), had lower odds of complying with their referral between 1 and 3 months as compared to 1–30 days. The 65–74 age group showed a trend of higher odds of delaying compliance as compared to the 40–64 group, with significance appearing in the 183–366 day period (OR 2.55 [1.17–5.55]). When patients had depleted their deductibles, they also had lower odds to be non-compliant (OR 0.12 [0.02–0.58]), as compared to being compliant between 1 and 30 days. The quarter in which patients were referred also significantly impacted compliance, with higher OR for Q2 in months 1–3 (OR 1.50 [1–2.25]) and lower ORs for Q2 (OR 0.43 [0.18–0.99]) and Q3 (OR 0.18 [0.05–0.62]) in months 3–6. Distance to the nearest hospital had odds close to 1 in all periods, indicating little to no impact on compliance. Females generally showed slightly higher odds of delaying compliance as compared to males across all periods, but none of the estimates were significant.

**Table 3:** Multilevel logistic regression for hospital or ICU admission in patients referred between 2015–2018

	Hospital admission	ICU admission
	OR [95% CI]	OR [95% CI]
Compliance period (ref: between 1 and 30 days)		
Between 1–3 months	1.23 [0.90, 1.68]	2.55 [1.20, 5.41]*
Between 3–6 months	0.97 [0.50, 1.89]	0.81 [0.10, 6.68]
Between 6–12 months	1.14 [0.59, 2.22]	0.66 [0.08, 5.49]
Age (ref: 40–64)		
65–74	1.79 [1.26, 2.54]**	2.41 [1.05, 5.52]*
75+	1.75 [1.20, 2.57]**	0.23 [0.05, 1.12]
Female	1.19 [0.89, 1.60]	1.87 [0.86, 4.07]
Neighborhood SES (ref: low)		
Medium-low	1.45 [0.89, 2.35]	0.87 [0.29, 2.61]
Medium-high	1.24 [0.75, 2.06]	1.13 [0.37, 3.48]
High	1.43 [0.84, 2.44]	0.36 [0.08, 1.59]
Comorbidities		
Mood or anxiety	0.94 [0.66, 1.36]	1.65 [0.73, 3.71]
Asthma	0.72 [0.51, 1.03]	0.30 [0.09, 1.03]
Cardiovascular risk factors	1.14 [0.84, 1.56]	1.64 [0.72, 3.69]
Diabetes type II	0.83 [0.57, 1.21]	1.27 [0.49, 3.32]
Osteoporosis	0.99 [0.65, 1.51]	0.91 [0.32, 2.64]
Distance to nearest hospital	1.00 [0.98, 1.02]	0.97 [0.92, 1.02]
Compliance period overlaps 1 st COVID-19 wave	0.71 [0.52, 0.99]*	0.53 [0.22, 1.31]

*ICU* Intensive Care Unit

*p < 0.05

**>p < 0.01

***p < 0.001

### Referral compliance and hospital admissions

The results of the multilevel logistic regression analysis are shown in in Table 3, depicting which factors were associated with hospital and ICU admissions. The compliance period was not significantly associated with hospital admissions for patients with COPD. However, age did significantly increases the odds, with individuals aged 65–74 and 75–84 having higher odds (OR = 1.79, 95% CI [1.26, 2.54] and OR = 1.75, 95% CI [1.20, 2.57], respectively) compared to those aged 40–64. For ICU admissions, there was an association with the compliance period, as patients who complied within 1–3 months had significantly higher odds of ICU admission (OR = 2.55, 95% CI [1.20, 5.41]) compared to those who complied earlier (between 1 and 30 days after referral). Age also influenced ICU admission, with individuals aged 65–74 showing significantly increased odds (OR = 2.41, 95% CI [1.05, 5.52]), as compared to individuals aged 40–64.

### Patients at high risk of cardiovascular disease

The study population for patients at high risk of cardiovascular disease consisted of 130,540 patient years between 2015 and 2018 (Table 4). Most patients were between 46 and 74 years old (71.2%), and 58.0% was female. Neighborhood SES was relatively low, and most patients lived in neighborhoods with low (30.3%) or medium-low (27.3%) socioeconomic status.

**Table 4:** Characteristics of prevalent patients at high risk of cardiovascular disease referred between 2015–2018, by referral status

	Non-referred (*N* = 128,346)	Referred (*N* = 2194)	Overall (*N* = 130,540)
Age in years (categorical)			
18–46	6390 (5.0%)	218 (9.9%)	6608 (5.1%)
46–64	50,280 (39.2%)	995 (45.4%)	51,275 (39.3%)
65–74	40,956 (31.9%)	635 (28.9%)	41,591 (31.9%)
75+	30,720 (23.9%)	346 (15.8%)	31,066 (23.8%)
Female	74,418 (58.0%)	1337 (60.9%)	75,755 (58.0%)
Socioeconomic status (SES)			
Low	38,923 (30.3%)	693 (31.6%)	39,616 (30.3%)
Medium-low	35,085 (27.3%)	586 (26.7%)	35,671 (27.3%)
Medium-high	29,749 (23.2%)	512 (23.3%)	30,261 (23.2%)
High	24,589 (19.2%)	403 (18.4%)	24,992 (19.1%)
Deductibles depleted	89,333 (69.6%)	1763 (80.4%)	91,096 (69.8%)
Comorbidities			
Mood or anxiety	17,972 (14.0%)	406 (18.5%)	18,378 (14.1%)
Other psychological complaints	2025 (1.6%)	40 (1.8%)	2065 (1.6%)
Dementia	1905 (1.5%)	10 (0.5%)	1915 (1.5%)
Diabetes type II	31,787 (24.8%)	421 (19.2%)	32,208 (24.7%)
Migraine	2938 (2.3%)	86 (3.9%)	3024 (2.3%)
Cardiac arrhythmia	10,456 (8.1%)	152 (6.9%)	10,608 (8.1%)
COPD	9645 (7.5%)	146 (6.7%)	9791 (7.5%)
Heart valve disorder	2798 (2.2%)	56 (2.6%)	2854 (2.2%)
Thyroid disorder	9265 (7.2%)	182 (8.3%)	9447 (7.2%)
Gout	10,670 (8.3%)	156 (7.1%)	10,826 (8.3%)
Rheumatoid arthritis	4341 (3.4%)	77 (3.5%)	4418 (3.4%)
Cancer	6390 (5.0%)	218 (9.9%)	6608 (5.1%)

### Referrals

Between 2015 and 2018, a total of 2194 (1.7%) existing patients at high risk of cardiovascular disease were referred and 128,346 (98.3%) were not referred (Table 4). Referred and non-referred patients mostly differed in age, where referred patients were more often in the working age, between 18 and 64 years. Patients living in neighborhoods with low socioeconomic status formed a substantial proportion of our study population, but no major differences were observed between referred and non-referred patients. A substantially (80.4% vs. 69.6%) higher percentage of referred patients had depleted their deductibles in the year of referral (excluding the medical specialist care for which they were referred – see method section). Mood- or anxiety disorders were more prevalent among referred patients. Diabetes mellitus type II, dementia and cancer were more prevalent in the non-referred patients.

**Table 5:** Characteristics and healthcare use of patients at high risk of cardiovascular disease referred between 2015–2018

	Immediate	Between 1–30 days	Between 1–3 months	Between 3–6 months	Between 6–12 months	> 1 year or non-compliant		Overall
Number of patients (compliance rate)	274 (12.4%)	1293 (58.9%)	360 (16.4%)	37 (1.7%)	37 (1.7%)	193 (8.8%)		2194
Age in years (categorical)								
18–46	25 (9.1%)	115 (8.9%)	50 (13.9%)	5 (13.5%)	5 (13.5%)		18 (9.3%)	218 (9.9%)
46–64	114 (41.6%)	608 (47.0%)	165 (45.8%)*-	15 (40.5%)	12 (32.4%)*-		81 (42.0%)	995 (45.4%)
65–74	82 (29.9%)	377 (29.2%)	101 (28.1%)*-	9 (24.3%)	11 (29.7%)		55 (28.5%)	635 (28.9%)
75+	53 (19.3%)	193 (14.9%)	44 (12.2%)*-	8 (21.6%)	9 (24.3%)		39 (20.2%)	346 (15.8%)
Female	170 (62.0%)	798 (61.7%)	209 (58.1%)	20 (54.1%)	21 (56.8%)		119 (61.7%)	1337 (60.9%)
Socioeconomic status (SES)								
Low	89 (32.5%)	383 (29.6%)	128 (35.6%)	14 (37.8%)	10 (27.0%)		69 (35.8%)	693 (31.6%)
Medium-low	70 (25.5%)	375 (29.0%)	71 (19.7%)*-	8 (21.6%)	9 (24.3%)		53 (27.5%)	586 (26.7%)
Medium-high	73 (26.6%)	310 (24.0%)	81 (22.5%)*-	7 (18.9%)	8 (21.6%)		33 (17.1%)	512 (23.3%)
High	42 (15.3%)	225 (17.4%)	80 (22.2%)	8 (21.6%)	10 (27.0%)		38 (19.7%)	403 (18.4%)
Deductibles depleted	238 (86.9%)	1004 (77.6%)	281 (78.1%)	33 (89.2%)	32 (86.5%)		175 (90.7%)*+	1763 (80.4%)
Quarter of referral								
Q1 (January – March)	70 (25.5%)	416 (32.2%)	81 (22.5%)	7 (18.9%)	11 (29.7%)		58 (30.1%)	643 (29.3%)
Q2 (April – June)	64 (23.4%)	259 (20.0%)	86 (23.9%)*+	9 (24.3%)	8 (21.6%)		45 (23.3%)	471 (21.5%)
Q3 (July – September	76 (27.7%)	264 (20.4%)	68 (18.9%)*+	11 (29.7%)*+	8 (21.6%)		42 (21.8%)	469 (21.4%)
Q4 (October – December)	64 (23.4%)	354 (27.4%)	125 (34.7%)	10 (27.0%)	10 (27.0%)		48 (24.9%)	611 (27.8%)
Distance to nearest hospital in kilometres	8.3 (6.6)	8.8 (7.8)	8.7 (7.8)	8.2 (7.7)	7.0 (6.8)		7.9 (7.8)	8.6 (7.6)
Comorbidities								
Mood or anxiety	61 (22.3%)	240 (18.6%)	62 (17.2%)	5 (13.5%)	6 (16.2%)		32 (16.6%)	406 (18.5%)
Other psychological complaints	9 (3.3%)	23 (1.8%)	4 (1.1%)	1 (2.7%)	1 (2.7%)		2 (1.0%)	40 (1.8%)
Dementia	0 (0%)	6 (0.5%)	2 (0.6%)	0 (0%)	0 (0%)		2 (1.0%)	10 (0.5%)
Diabetes type II	53 (19.3%)	254 (19.6%)	63 (17.5%)	6 (16.2%)	7 (18.9%)		38 (19.7%)	421 (19.2%)
Migraine	8 (2.9%)	57 (4.4%)	9 (2.5%)	1 (2.7%)	1 (2.7%)		10 (5.2%)	86 (3.9%)
Cardiac arrhythmia	27 (9.9%)	72 (5.6%)	34 (9.4%)*+	5 (13.5%)	9 (24.3%)*+		5 (2.6%)	152 (6.9%)
COPD	15 (5.5%)	87 (6.7%)	18 (5.0%)	3 (8.1%)	5 (13.5%)		18 (9.3%)	146 (6.7%)
Heart valve disorder	8 (2.9%)	30 (2.3%)	9 (2.5%)	4 (10.8%)*+	1 (2.7%)		4 (2.1%)	56 (2.6%)
Thyroid disorder	25 (9.1%)	96 (7.4%)	28 (7.8%)	5 (13.5%)	3 (8.1%)		25 (13.0%)*+	182 (8.3%)
Gout	21 (7.7%)	91 (7.0%)	21 (5.8%)	6 (16.2%)	6 (16.2%)		11 (5.7%)	156 (7.1%)
Rheumatoid arthritis	11 (4.0%)	49 (3.8%)	10 (2.8%)	1 (2.7%)	1 (2.7%)		5 (2.6%)	77 (3.5%)
Cancer	36 (13.1%)	186 (14.4%)	63 (17.5%)*+	9 (24.3%)	9 (24.3%)		44 (22.8%)*+	347 (15.8%)
Kidney	8 (6.6%)	33 (2.6%)	11 (3.1%)	1 (2.7%)	1 (2.7%)		7 (3.6%)	61 (2.8%)
Specialist consultations < 2 year after referral								
Diagnostics	261 (95.3%)	1206 (93.3%)	302 (83.9%)	27 (73.0%)	28 (75.7%)		25 (13.0%)	1849 (84.3%)
Admission	66 (24.1%)	83 (6.4%)	24 (6.7%)	6 (16.2%)	3 (8.1%)		5 (2.6%)	187 (8.5%)
* Short (0–5 days)*	52 (19.0%)	53 (4.1%)	16 (4.4%)	5 (13.5%)	3 (8.1%)		4 (2.1%)	133 (6.1%)
* Moderate (5–28 days)*	13 (4.7%)	27 (2.1%)	7 (1.9%)	0 (0%)	0 (0%)		0 (0%)	47 (2.1%)
* Long (> 28 days)*	1 (0.4%)	3 (0.2%)	1 (0.3%)	1 (2.7%)	0 (0%)		1 (0.5%)	7 (0.3%)
ICU admission	3 (1.1%)	6 (0.5%)	4 (1.1%)	0 (0%)	0 (0%)		0 (0%)	13 (0.6%)
* Short (0–5 days)*	2 (0.7%)	5 (0.4%)	4 (1.1%)	0 (0%)	0 (0%)		0 (0%)	11 (0.5%)
* Moderate (5–28 days)*	1 (0.4%)	1 (0.1%)	0 (0%)	0 (0%)	0 (0%)		0 (0%)	2 (0.1%)
* Long (> 28 days)*	0 (0%)	0 (0%)	0 (0%)	0 (0%)	0 (0%)		0 (0%)	0 (0%)
Surgery	6 (2.2%)	24 (1.9%)	11 (3.1%)	1 (2.7%)	0 (0%)		0 (0%)	42 (1.9%)
Day treatment	77 (28.1%)	164 (12.7%)	43 (11.9%)	3 (8.1%)	5 (13.5%)		2 (1.0%)	294 (13.4%)

*negatively (-) or positively (+) associated with complying in the respective referral period (ref: 1–30 days), based on the multinominal regression analyses as shown in Additional File V

### Referral compliance and associated factors

In 12.4% of the referrals, patients complied immediately on the day of referral, suggesting the need for acute care (Table 5). Between 1 and 30 days after referral, the compliance rate was 58.9%. After 3 months, most patients had complied with their referral (87.8%). Few more patients complied between 3 and 12 months (3.4%). Ultimately, 8.8% of patients did not comply with their referral within 1 year.

Multinominal regression analyses (Additional File V, Table A5-2) showed that different factors were significantly associated with time to referral compliance for patients at high risk of CVD. In these multinomial regression analyses, patients who complied within 1–30 days after the referral date served as the reference group. Patients who complied immediately (on the same day) were excluded due to the likelihood of acute care needs. Older age groups (46–64, 65–74, and 75+) consistently showed reduced odds of delaying referral compliance compared to the 18–45 group. This is significant in the 1–3 month period for patients aged 46–64 (OR 0.62 [0.42–0.92]), 65–74 (OR 0.56 [0.36–0.87]), or 75+ (OR 0.47 [0.28–0.79]). Longer compliance delay is also less likely for these age groups, although this is statistically insignificant. Females showed slightly lower odds of delaying compliance as compared to males across all periods, but none of the estimates were significant. Patients with lower neighborhood SES had lower odds of delaying compliance, with significant differences between medium-low and medium-high neighborhood SES (OR 0.52 [0.36–0.75] and OR 0.69 [0.48–1.00], respectively) between 1 and 3 months. Patients with cardiac arrhythmia had higher odds of complying later than 1–30 days, respectively (OR 2.01 [1.28–3.14]) for 1–3 months, and (OR 4.64 [1.95–11.04]) for 6–12 months. Patients with cancer had higher odds of complying between 1 and 3 months as compared to 1–30 days (OR 1.51 [1.09–2.11]). Heart valve disorders (OR 5.68 [1.76–18.34]) increased the odds of complying respectively between 3 and 6 months. Thyroid disorder (OR 1.83 [1.12–2.98]) and cancer (OR 1.62 [1.10–2.4]) were associated with higher odds of non-compliancy. Surprisingly, depletion of deductibles was also associated with higher odds of non-compliancy (OR 2.77 [1.65–4.65]). Referral timing also influenced compliance. Patients referred after Q1 (January – March) were more likely to delay on their compliance for one month or more. This is significant for 1–3 months for patients referred in Q2 (OR 1.75 1.22–2.49]) and Q4 (OR 1.92 [1.39–2.66]). For 3–6 months this is significant in Q3 (OR 2.7[1.00–7.34]).

### Referral compliance and hospital admissions

Among patients requiring acute care, 24.1% was admitted to the hospital, and 1.1% got admitted to the intensive care (Table 5). Hospital admittance fell sharply for referrals complied with between 1 and 30 days, but increased again when compliance occurred after 3–6 months. For patients that did not immediately comply with their referral, the regression output in Table 6 shows factors associated with hospital admission. As was the case with COPD, referral compliance period was not significantly associated with hospital admissions for patients at high risk of cardiovascular disease. Age however, showed a strong association, with older individuals having significantly higher odds of hospital admission. Being female was associated with reduced odds of hospital admission (OR 0.65 [0.42–1.00]). High neighborhood SES was associated with lower odds of hospital admission (OR 0.43 [0.20–0.93]). Patients that had also been diagnosed with cardiac arrhythmia (OR 3.43 [1.94–6.06]) and heart valve disorder (OR 2.66 [1.08–6.59]), had significantly higher odds of hospital admission. The distance to the nearest hospital did not appear to influence hospital admissions.

Regression analyses for ICU admissions were not feasible due to the low number of ICU admissions among patients at high risk for CVD (*N* = 13).

**Table 6:** Multilevel logistic regression for hospital admission in patients referred between 2015–2018

	Hospital admission
	OR [95% CI]
Compliance period (ref: between 1–30 days)	
Between 1–3 months	0.91 [0.54, 1.54]
Between 3–6 months	2.07 [0.74, 5.76]
Between 6–12 months	0.89 [0.24, 3.33]
Age(ref: 40–64)	
46–64	2.04 [0.75, 5.53]
65–74	1.63 [0.57, 4.68]
75+	3.53 [1.19, 10.49]*
Female	0.65 [0.42, 1.00]*
Neighborhood SES (ref: low)	
Medium-low	0.98 [0.56, 1.70]
Medium-high	0.95 [0.54, 1.66]
High	0.43 [0.20, 0.93]*
Comorbidities	
Mood or anxiety	0.98 [0.56, 1.72]
Other psychological complaints	1.25 [0.26, 6.04]
Dementia	1.06 [0.11, 10.56]
Diabetes type II	1.46 [0.92, 2.34]
Migraine	0.60 [0.14, 2.69]
Cardiac arrhythmia	3.43 [1.94, 6.06]***
COPD	1.53 [0.78, 3.01]
Heart valve disorder	2.66 [1.08, 6.59]*
Thyroid disorder	1.51 [0.77, 2.98]
Gout	1.11 [0.55, 2.24]
Rheumatoid arthritis	1.08 [0.39, 2.99]
Cancer	0.83 [0.47, 1.48]
Kidney	1.83 [0.65, 5.17]
Distance to nearest hospital	0.94 [0.75, 1.18]
Compliance period overlaps 1 st COVID-19 wave	0.57 [0.35, 0.93]*

Regression analyses for ICU admissions were not feasible due to the low number of ICU admissions among patients at high risk for CVD (*N* = 13)

*p < 0.05

**p < 0.01

***p < 0.001

## Discussion

The aims of this study were: (1) to gain insight into which patients with COPD and at high risk of cardiovascular diseases are referred to medical specialist care, (2) to gain deeper insight into specialty referral compliance and associated factors for these patients, and (3) to gain insight into the association between time to specialty referral compliance and hospital admissions. We found that only a small proportion of all prevalent COPD (3.1%) and high-risk CVD (1.7%) patients were referred to specialist care. Among those referred, respectively 13.7% and 12.4% immediate comply on the day of referral, suggesting the need for urgent care. Compliance then increased steadily over time, with most patients complying within three months. Within 6 months 90.9% of COPD patients and 89.5% of CVD patients had complied with their referral. van Dijk et al. (2016) reported similar numbers, even though they reported on ICPC chapter and not individual diagnoses. They found a compliance rate of 90.5% for the respiratory chapter and 88.3% for the circulatory chapter. Despite these high rates, after more than a year a notable proportion (5.2% of COPD patients and 8.8% of CVD patients) still had not complied with their referral.

Several factors were associated with time to referral compliance. Depletion of deductibles is one of these factors. Patient with COPD who had depleted their deductibles, were less likely to delay on their compliance. Also, they were less likely to be non-compliant overall, suggesting that deductibles could form a barrier for referral compliance among COPD patients. This is in line with earlier research [5]. Patients with COPD referred at the start of the year were more likely to delay compliance with their referral. Since deductibles reset annually, this could possibly be due to unmet deductibles. Surprisingly however, patients at high risk of CVD who had depleted their deductibles, actually were more likely to be non-compliant. This is contrary to earlier research on deductibles [22, 23]and seems counterinitiative. When considering alternative explanations, it might be possible that these patients have depleted their deductibles in another medical specialism than cardiology. This would also explain their non-compliancy, as they might get sufficient treatment elsewhere, under an existing DRG episode. Treatment at another specialism might also be more likely for patients at high risk for CVD than for COPD patients, since cardiovascular risk factors are less specific than COPD. However, the trend in the association with the quarter of referral is also less clear for this patient group, since a higher likelihood of complying can be seen for patients referred between Q2-Q4 across all compliance periods. However, this association was again not significant.

Another factor that is associated with time to referral compliance is age. Patients with COPD aged 65–74, are more likely to comply within 6–12 months as compared to the youngest group aged 40–64 years old. In other words, they delay on their referral compliance. This is only found for patients aged 65–74, and not for patients of 75 years and older. It might be that patients aged 65–74 still perceive their health to be relatively stable or manageable, which leads them to postpone specialist care [24–27]. Factors such as denial or avoidance might be most pronounced in this age group, if patients are not willing to face their worsening medical condition [24–27]. Patients at high risk of CVD however, show different behavior. Older patients, aged 46 years and over, are less likely to comply 1 month after their referral or later. While this reduction in odds is insignificant for 3 months or later due to low numbers, it is significant within 1–3 months. A possible explanation could be the difference in disease mechanism as compared to COPD [28, 29]. While the progression of COPD may be more subtle at first, CVD risk factors might induce fear for acute events like a heart attack or stroke. As a result, patients may potentially feel a heightened sense of urgency, recognizing that a delay in compliance could result in severe outcomes. Family history, which plays a major role in CVD risk factors, could potentially enforce this [20].

When looking at comorbidities for patients with COPD, patients with additional CVD risk factors were more likely to comply within the first month of their referral. This suggests that the presence of CVD may create a greater sense of urgency for timely compliance. When looking at other comorbidities, COPD patients that have mood or anxiety disorders, are more likely to be non-compliant. This is well-known from the scientific literature [30]. For patients at high risk of cardiovascular diseases, cardiac arrhythmia seems to play a major role. Cardiac arrhythmia increases the likelihood of postponing referral compliance to 1 month after referral or more. Unlike conditions such as acute myocardial infarction or stroke, cardiac arrhythmias may not always result in immediate life-threatening consequences. Therefore, patients might not always perceive a referral to be urgently necessary and could potentially delay their visit to a specialist. For some, the fear of diagnosis or potential treatments (like surgery or pacemaker implantation) may cause them to delay compliance. Last but not least, having cancer is also associated with longer time to referral compliance for patients at high risk of CVD. These patients comply later (between 1 and 3 months after referral). Since cancer patients often have intensive and time-consuming treatment schedules, including chemotherapy, radiation, or surgery, CVD referrals may initially not be prioritized. However, CVD patients with comorbid cancer are also more likely to not comply at all. Similar to CVD patients that depleted their deductible, not complying to a cardiologist referral could be due to existing treatment for CVD at another specialism, in this case likely the oncologist. When patients have chemotherapy-induced cardiotoxicity for example, the principal physician could still be the oncologist.

Time to specialty referral compliance did not seem to be associated with hospital admission, neither for patients with COPD nor patients at high risk of CVD. However, referral compliance between 1 and 3 months did seem to be associated with ICU admissions for patients with COPD, as compared to patients complying within 1 month. This suggests that delaying referral compliance for COPD patients could potentially have adverse effects. We also found that COPD patients that have depleted their deductibles are less likely to be non-compliant. In other words, patients that still have to pay deductibles, are more likely to be non-compliant. This suggests that deductibles could form a barrier for referral compliance among COPD patients, which might have negative consequences for this patient group.

### Strengths and limitations

We are the first to study referral compliance at a disease specific level, to our knowledge. Moreover, no studies in the Netherlands have linked depletion of deductibles to referral compliance behavior on individual level before. The use of combined nationwide routine healthcare data enabled us to analyze a large cohort of patients with chronic diseases, studying their referral compliance for two years after referral over several healthcare providers across multiple consecutive years. This extensive dataset allowed us to comprehensively map healthcare utilization patterns, as well as the relationship between specialty referral compliance and specialist care utilization. Despite this large dataset, certain analyses were conducted on a relatively small sample. Since only a small proportion of patients was referred to specialist care, even less patients complied with these referrals and then an even smaller subset experienced events such as ICU admission. This reduces the power of the analyses and hence the conclusions that can be drawn. Additionally, since our study had a two year follow-up period after referral, we cannot assess healthcare utilization beyond this timeframe. However, we assume that after two years, the clinical context of the referral changes significantly. Another limitation was the inability to definitively determine whether delays in compliance were due to intentional patient decisions or external factors like long waiting times or personal circumstances. While we excluded immediate compliance on the referral date to avoid confounding by acute care needs, it is possible that some patients in the reference group also required acute care. However, the observed large difference in compliance on the referral date as compared to two or three days after the referral date supports our choice of this cutoff point (see Additional File II). Also, there is a possibility that some patients received care in other specialties, particularly for patients at high risk of CVD. This could have resulted in an overestimation of the non-compliance rates reported in this study. Moreover, it is not known whether all the admissions for patients requiring acute care were due to acute exacerbation of COPD (AECOPD), as this information is not included in the hospital DRG data available for this study. However, it is highly likely that this was indeed the reason for admission. Patients that immediately comply with their referral and are not admitted, undergo for instance diagnostic consultations (95.3%) or day treatments (28.1%). Patients that are indeed admitted are in need of more intensive treatment, which is likely due to exacerbation. Since they are admitted on the day of their referral, the nature of the admittance is assumed to be acute and related to AECOPD. Lastly, we could only use neighborhood socioeconomic status (SES) data in this study, rather than individual SES. This limitation may have led to an underestimation of the influence of SES on referral compliance.

### Recommendations for future studies

For patients with COPD, it may be valuable to investigate their perspectives on how deductibles influence their referral compliance behavior. This could for example be studied through focus groups or interview studies. The study population could either be patients under GP treatment that might need specialist care in the future, or COPD patients that had to be admitted to the ICU in the past. If these patients respectively state that the deductible would hinder them or has hindered them in the past from seeking appropriate care, then policy intervention might be useful.

Ultimately, 5.2% of COPD patients and 8.8% of patients at high risk of CVD, did not comply with their specialist referral. Future research could examine whether patients that do not comply with specialty referrals have worse health status, or whether the health issue has been managed appropriately without the need for medical specialist care.

### Conclusion and policy implications

Our study shows that while many patients comply with their referral, compliance delay or non-compliance is present and can be associated with ICU admissions in COPD patients. There are important differences in referral compliance among patients with COPD and patients at high risk of CVD. Factors such as deductibles, age, and comorbidities influence the timing and likelihood of specialty referral compliance. While most patients complied within three months, a small percentage remains non-compliant even after a year, raising concerns about potential health risks. Delayed compliance in COPD patients is significantly associated with ICU admission within the 1–3 month period compared to the 1–30 day reference period. This association is not observed in later periods (3–6 or 6–12 months), which may reflect differences in patient behavior or disease progression during the early stages of referral. Further research is needed to explore the underlying mechanisms. Policy interventions could encourage compliance by addressing financial barriers such as deductibles, which seem to discourage compliance among COPD patients.

## Supplementary Information

Supplementary Material Additional File I.docx. Title of data: ICPC, DRG and ATC codes. Description of data: clinical codes corresponding with the selection criteria of our study sample. Additional File II.docx. Title of data: referral- and medical specialist codes. Description of data: codes for diagnoses, referral and medical specialist care used for referral- and referral adherence rates. Additional File III.docx. Title of data: time to referral compliance. Description of data: time between referral date and start of hospital treatment episode.Additional File IV.docx.Title of data: time in dataset. Description of data: Years in dataset for patients in our study sample. Additional File V.docx. Title of data: multinominal logistic regression output. Description of data: Multinominal logistic regression for factors associated with compliance period.

## Data Availability

The data that support the conclusions of this study are available from Nivel and the NHCI, but restrictions apply to the availability of these data, which were used under license for the current study and so are not publicly available. According to the GDPR, the National Health Care Institute has a legal basis to process this health data. Datasets are only available for organizations with a legal basis to process this health data which is in accordance with the legal basis for which the National Health Care Institute has collected the data. Data at the aggregated level are available from the corresponding author upon request and with the permission of Nivel and the NHCI.

## Abbreviations

ATC: Anatomical Therapeutic Chemical
COPD: Chronic Obstructive Pulmonary Disease
CVD: Cardiovascular Disease
DRG: Diagnosis-Related Group
EHR: Electronic Health Records
GP: General Practitioner
ICU: Intensive Care Unit
ICPC: International Classification of Primary Care
NHCI: National Healthcare Institute
OR: Odds Ratio
PCD: Primary Care Database
Q: Quarter
SES: Socioeconomic Status

## Footnotes

1 Patients with an ICPC record in EHR data for angina pectoris (K74), acute myocardial infarction (K75), Ischemic heart disease (K76), transient cerebral ischemia (K89), stroke or cerebrovascular accident (K90) or atherosclerosis excluding heart and brain (K91) were flagged as having experienced a cardiovascular event.
