# Supplementary material for: Referral compliance and subsequent hospital admissions for COPD and cardiovascular disease in the Netherlands: a data linkage study
Source: BMC Health Serv Res. 2025 Oct 9;25:1342. doi: 10.1186/s12913-025-13391-4 (PMC12512944; doi:10.1186/s12913-025-13391-4)
Supplement: Supplementary file 1 — Supplementary Material Additional File I.docx. Title of data: ICPC, DRG and ATC codes. Description of data: clinical codes corresponding with the selection criteria of our study sample. Additional File II.docx. Title of data: referral- and medical specialist codes. Description of data: codes for diagnoses, referral and medical specialist care used for referral- and referral adherence rates. Additional File III.docx. Title of data: time to referral compliance. Description of data: time between referral date and start of hospital treatment episode.Additional File IV.docx.Title of data: time in dataset. Description of data: Years in dataset for patients in our study sample. Additional File V.docx. Title of data: multinominal logistic regression output. Description of data: Multinominal logistic regression for factors associated with compliance period. [file 12913_2025_13391_MOESM1_ESM.docx]

**Additional File I: ICPC, DRG and ATC codes**

Table A1-1. ICPC-codes and DRG-codes for patients at high cardiovascular risk.

| **ATC- /ICPC- / DRG-code** | **Description** |
| --- | --- |
| **ATC-code** |  |
| C02 | Antihypertensives |
| C03 | Diuretics |
| C07 | Beta-blockers |
| C08 | Calcium channel blockers |
| C09 | Agents acting on the renin-angiotensin system |
| C10 | Lipid-lowering agents |
|  |  |
| **ICPC-code** |  |
| K86 | Hypertension without organ damage |
| K87 | Hypertension with organ damage |
| T93 | Lipid metabolism disorder(s) |
|  |  |
| **DRG-code** |  |
| 320-902 | Cardiology – Hypertension |
| 313-311 | Internal Medicine – Hypertension |
| 316-4003 | Pediatrics – Hypertension |
| 318-901 | Gastroenterology – Hypertension |
| 320-901 | Cardiology – Lipid metabolism disorders |
| 313-271 | Internal Medicine – Primary dyslipidemia |
| 313-133 | Internal Medicine – Chronic care for patients with multiple cardiovascular risk factors |
| 320-905 | Cardiology – Chronic care for patients with multiple cardiovascular risk factors |

Table A1-2. ICPC-codes and DRG-codes for chronic obstructive pulmonary disease.

| **ICPC- / DRG-code** | **Description** |
| --- | --- |
| **ICPC-code** |  |
| R95 | Emphysema / COPD |
|  |  |
| **DRG-code** |  |
| 0322-1241 | Pulmonology - COPD |
| 0335-0272 | Clinical geriatrician - COPD |

Table A1-3. DRG-codes for kidney dialysis and kidney- or heart transplant.

| DRG-code | Description |
| --- | --- |
| 320-903 | Cardiology - Guidance for heart transplantation |
| 320-904 | Cardiology - Guidance for heart-lung transplantation |
| 328-2910 | Cardiothoracic Surgery - Heart transplantation |
| 328-2930 | Cardiothoracic Surgery - Heart-lung transplantation |
| 328-2940 | Cardiothoracic Surgery - Long-term ventricular assist device implantation |
| 328-2430 | Cardiothoracic Surgery - Short-term ventricular assist device |
| 322-2202 | Pulmonology - Guidance for heart-lung transplantation |
| 313-326 | Internal Medicine - Chronic hemodialysis in hospital (passive) |
| 313-327 | Internal Medicine - Chronic hemodialysis in an independent dialysis center |
| 313-328 | Internal Medicine - Chronic hemodialysis at home |
| 313-331 | Internal Medicine - Continuous ambulatory peritoneal dialysis (CAPD) |
| 313-332 | Internal Medicine - Automated peritoneal dialysis (APD) |
| 313-333 | Internal Medicine - Chronic hemodialysis in self-treatment center (active) |
| 313-334 | Internal Medicine - Chronic hemodialysis in self-treatment center (training) |
| 313-335 | Internal Medicine - Chronic hemodialysis in self-treatment center (passive) |
| 313-336 | Internal Medicine - Chronic hemodialysis at home |
| 313-337 | Internal Medicine - Chronic hemodialysis at home with nursing support |
| 313-338 | Internal Medicine - Chronic hemodialysis at home (night) |
| 313-339 | Internal Medicine - Chronic hemodialysis in an institution |
| 303-501 | Surgery - Kidney transplantation |
| 303-557 | Surgery - Kidney transplantation recipient |
| 313-76 | Internal Medicine - Guidance for kidney transplantation recipient |
| 313-341 | Internal Medicine - Kidney transplantation |
| 313-342 | Internal Medicine - Pre-transplantation screening (recipient) |
| 313-344 | Internal Medicine - Kidney transplantation ≤ 365 days |
| 313-346 | Internal Medicine - Kidney transplantation > 365 days |
| 316-7907 | Pediatrics - Guidance for kidney transplantation recipient |
| 362-225 | Radiology - Native dialysis shunt |
| 362-226 | Radiology - Synthetic dialysis shunt |
| 303-435 | Surgery - Shunt surgery including revision for kidney disease |
| 313-81 | Internal Medicine - Liver and kidney transplantation pathway (recipient) |
| 303-561 | Surgery - Liver and kidney transplantation (recipient) |
| 328-2270 | Cardiothoracic Surgery - Dialysis catheter placement |

**Additional File II: referral- and medical specialist codes**

Table A2-1. Codes for diagnoses (ICPC), referral and medical specialist care used for referral- and referral adherence rates for patients at high risk for cardiovascular diseases.

| Codes | Description |
| --- | --- |
| ICPC-code |  |
| K01 | Pain of the heart |
| K02 | Pressure of the heart |
| K03 | Other heart- or vascular pain |
| K74 | Angina pectoris |
| K75 | Acute myocard infarction |
| K76 | Ischaemic heart diseases |
| K86 | Hypertension without organ failure |
| K87 | Hypertension with organ failure |
| K89 | TIA |
| K90 | CVA |
| K91 | Atherosclerosis |
| T93 | Fat metabolism disorder(s) |
|  |  |
| Reason for referral |  |
|  | Ischaemic heart diseases |
|  | Cerebrovascular diseases |
|  | Cardio-vascular risk |
|  | Hypertension |
|  | Fat metabolism disorder(s) |
|  | Vascular care |
|  | Heart rehabilitation |
|  | Compelling medical history: hypertension |
|  |  |
| Specialism |  |
|  | Internal medicine |
|  | Pediatrics |
|  | Cardiology |
|  | Geriatrics |
|  | Surgery |
|  | Neurology |
|  | Rehabilitation medicine |

Table A2-2. Codes for diagnoses (ICPC), referral and medical specialist care used for referral- and referral adherence rates for patients with chronic obstructive pulmonary disease.

| Codes | Description |
| --- | --- |
| ICPC-code |  |
| R95 | COPD |
|  |  |
| Reason for referral |  |
|  | COPD |
|  |  |
| Specialism |  |
|  | Internal medicine |
|  | Geriatrics |
|  | Pulmonology |
|  | Rehabilitation medicine |

**Additional File III: time to referral compliance**


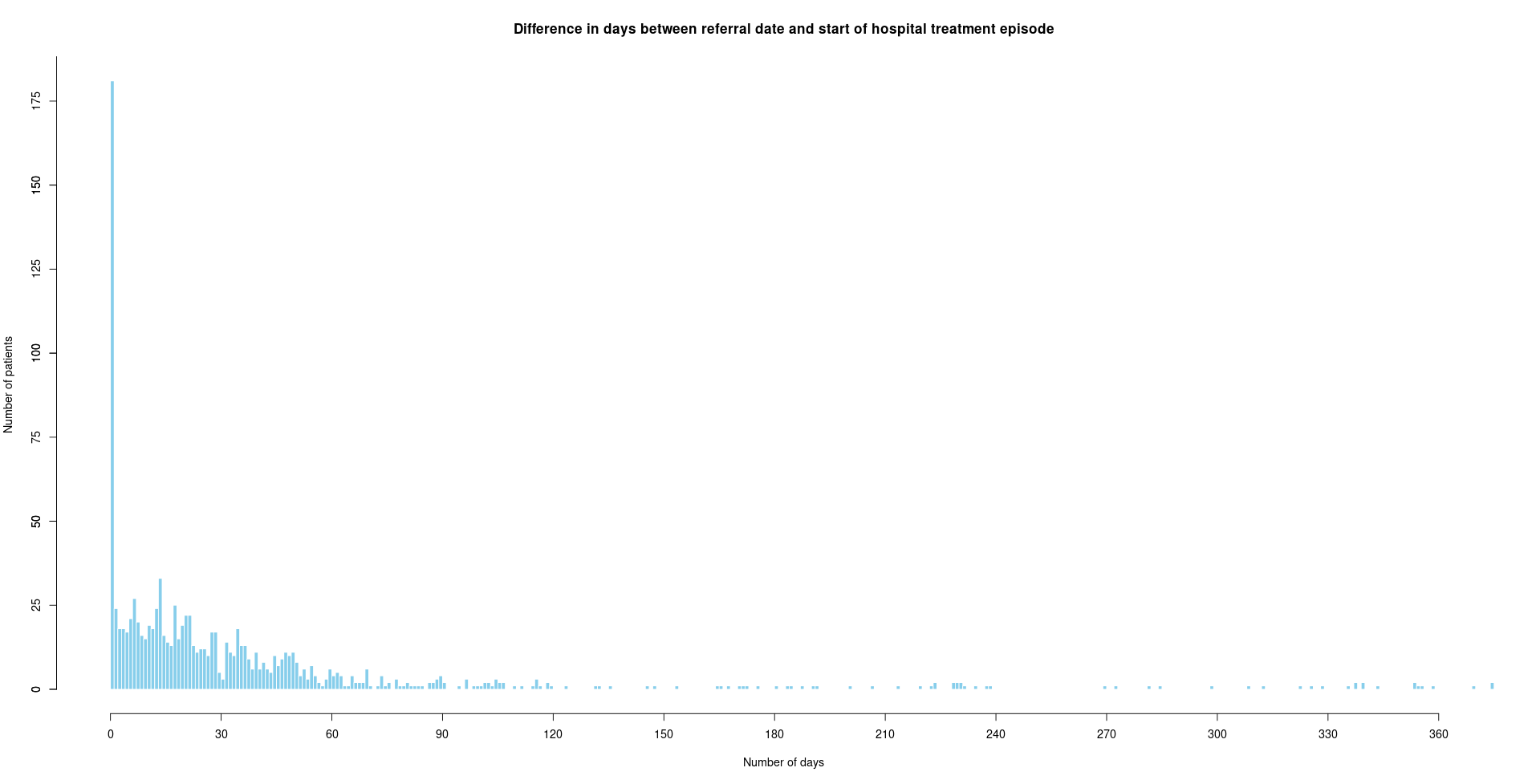


**Figure A3-1.** Difference in days between referral date and start of hospital treatment episode for patients with chronic obstructive pulmonary disease. Number of patients are shown per day, within one year after referral.


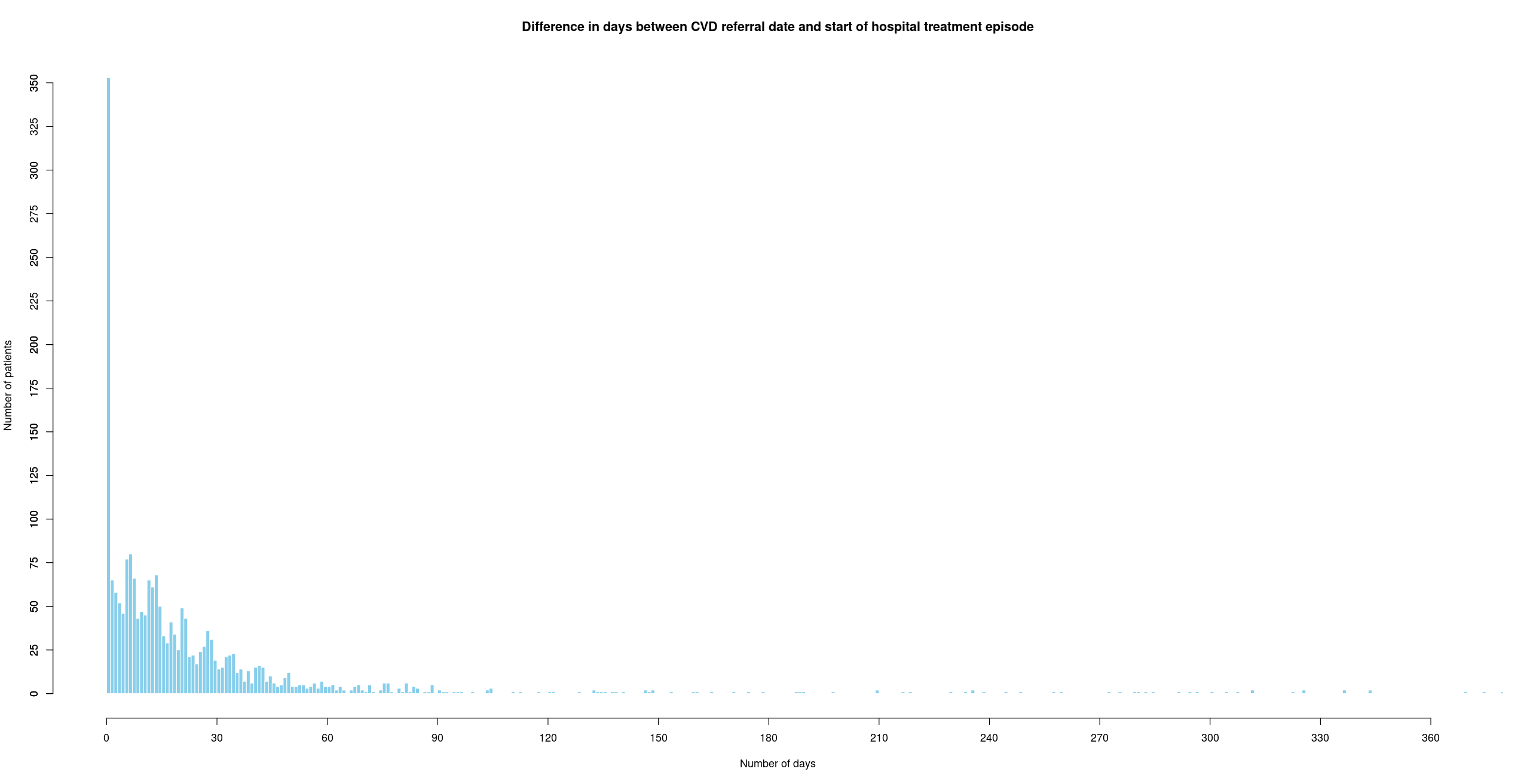


**Figure A3-2.** Difference in days between referral date and start of hospital treatment episode for patients at high risk of cardiovascular disease. Number of patients are shown per day, within one year after referral.

**Additional File IV: time in dataset**

Table A4-1. Years in dataset for patients with chronic obstructive pulmonary disease.

| Years in dataset | Number of patients |
| --- | --- |
| 1 | 7,005 |
| 2 | 4,714 |
| 3 | 3,643 |
| 4 | 2,061 |

Table A4-2. Years in dataset for patients with at high risk of cardiovascular disease.

| Years in dataset | Number of patients |
| --- | --- |
| 1 | 25,130 |
| 2 | 16,635 |
| 3 | 13,984 |
| 4 | 7,547 |

**Additional File V: multinominal logistic regression output**

**Table A5-1.** Multinominal logistic regression for factors associated with COPD compliance period

| **Compliance period** | **Variables** | **OR** | **CI 2.5%** | **CI 97.5%** |
| --- | --- | --- | --- | --- |
| **Between 31-90 days** | (Intercept) | 2.28 | 0.63 | 8.23 |
| Age (ref: 40-64) | 65-74 | 1.2 | 0.84 | 1.71 |
|  | 75+ | 1.03 | 0.69 | 1.52 |
| Gender (ref: male) | Female | 1.08 | 0.79 | 1.46 |
| Neighbourhood SES (ref: high) | Low | 0.86 | 0.54 | 1.37 |
|  | Medium-low | 0.82 | 0.5 | 1.34 |
|  | Medium-high | 0.74 | 0.43 | 1.25 |
| Deductibles depleted (ref: no) | Yes | 0.22* | 0.07 | 0.72 |
| Quarter of referral (ref: Q1) | Q2 (April – June) | 1.5* | 1 | 2.25 |
|  | Q3 (July – September | 1.43 | 0.94 | 2.19 |
|  | Q4 (October – December) | 1.14 | 0.73 | 1.77 |
| Comorbidities (ref: no) | Mood or anxiety disorder | 0.96 | 0.66 | 1.4 |
|  | Asthma | 1.04 | 0.73 | 1.48 |
|  | Cardiovascular diseases | 0.7* | 0.51 | 0.96 |
|  | Diabetes Mellitus Type II | 0.92 | 0.62 | 1.36 |
|  | Osteoporosis | 1.12 | 0.72 | 1.74 |
| Distance to nearest hospital | Distance in km | 1.01 | 0.99 | 1.02 |
| Overlap 1^st^ COVID-19 wave (ref: no) | Yes | 1.19 | 0.86 | 1.64 |
| **Between 91-182 days** | (Intercept) | 0.43 | 0.04 | 5.26 |
| Age (ref: 40-64) | 65-74 | 1.5 | 0.72 | 3.14 |
|  | 75+ | 0.88 | 0.36 | 2.12 |
| Gender (ref: male) | Female | 1 | 0.53 | 1.91 |
| Neighbourhood SES (ref: high) | Low | 0.9 | 0.31 | 2.64 |
|  | Medium-low | 1.06 | 0.35 | 3.19 |
|  | Medium-high | 1.6 | 0.53 | 4.85 |
| Deductibles depleted (ref: no) | Yes | 0.4 | 0.04 | 3.87 |
| Quarter of referral (ref: Q1) | Q2 (April – June) | 0.43* | 0.18 | 0.99 |
|  | Q3 (July – September | 0.18* | 0.05 | 0.62 |
|  | Q4 (October – December) | 0.74 | 0.35 | 1.59 |
| Comorbidities (ref: no) | Mood or anxiety disorder | 0.65 | 0.27 | 1.53 |
|  | Asthma | 1.39 | 0.68 | 2.85 |
|  | Cardiovascular diseases | 0.75 | 0.39 | 1.43 |
|  | Diabetes Mellitus Type II | 0.55 | 0.21 | 1.48 |
|  | Osteoporosis | 1.7 | 0.75 | 3.84 |
| Distance to nearest hospital | Distance in km | 0.97 | 0.93 | 1.01 |
| Overlap 1^st^ COVID-19 wave (ref: no) | Yes | 1.19 | 0.6 | 2.4 |
| **Between 183-366 days** | (Intercept) | 0.2 | 0.02 | 2.53 |
| Age (ref: 40-64) | 65-74 | 2.55* | 1.17 | 5.55 |
|  | 75+ | 1.18 | 0.45 | 3.07 |
| Gender (ref: male) | Female | 1.38 | 0.71 | 2.67 |
| Neighbourhood SES (ref: high) | Low | 1.46 | 0.47 | 4.53 |
|  | Medium-low | 1.33 | 0.41 | 4.4 |
|  | Medium-high | 1.18 | 0.33 | 4.15 |
| Deductibles depleted (ref: no) | Yes | 0.22 | 0.02 | 2.11 |
| Quarter of referral (ref: Q1) | Q2 (April – June) | 1.27 | 0.56 | 2.85 |
|  | Q3 (July – September | 0.94 | 0.37 | 2.41 |
|  | Q4 (October – December) | 0.91 | 0.35 | 2.32 |
| Comorbidities (ref: no) | Mood or anxiety disorder | 1.11 | 0.52 | 2.39 |
|  | Asthma | 1.43 | 0.7 | 2.92 |
|  | Cardiovascular diseases | 0.81 | 0.41 | 1.61 |
|  | Diabetes Mellitus Type II | 1.3 | 0.6 | 2.82 |
|  | Osteoporosis | 0.96 | 0.38 | 2.47 |
| Distance to nearest hospital | Distance in km | 0.98 | 0.94 | 1.02 |
| Overlap 1^st^ COVID-19 wave (ref: no) | Yes | 0.65 | 0.31 | 1.4 |
| **Non-compliant** | (Intercept) | 0.51 | 0.08 | 3.39 |
| Age (ref: 40-64) | 65-74 | 0.67 | 0.31 | 1.44 |
|  | 75+ | 1.48 | 0.73 | 2.98 |
| Gender (ref: male) | Female | 1.7 | 0.94 | 3.07 |
| Neighbourhood SES (ref: high) | Low | 1.24 | 0.48 | 3.21 |
|  | Medium-low | 1.14 | 0.42 | 3.11 |
|  | Medium-high | 0.92 | 0.31 | 2.78 |
| Deductibles depleted (ref: no) | Yes | 0.12* | 0.02 | 0.58 |
| Quarter of referral (ref: Q1) | Q2 (April – June) | 1.54 | 0.72 | 3.3 |
|  | Q3 (July – September | 1.06 | 0.45 | 2.49 |
|  | Q4 (October – December) | 1.6 | 0.72 | 3.57 |
| Comorbidities (ref: no) | Mood or anxiety disorder | 1.74 | 0.94 | 3.23 |
|  | Asthma | 0.99 | 0.51 | 1.95 |
|  | Cardiovascular diseases | 0.68 | 0.37 | 1.23 |
|  | Diabetes Mellitus Type II | 1.21 | 0.61 | 2.41 |
|  | Osteoporosis | 1.76 | 0.86 | 3.62 |
| Distance to nearest hospital | Distance in km | 0.98 | 0.94 | 1.02 |
| Overlap 1^st^ COVID-19 wave (ref: no) | Yes | 1.04 | 0.56 | 1.91 |

**Table A5-2.** Multinominal logistic regression for factors associated with CVD compliance period

| **Compliance period** | **Variables** | **OR** | **CI 2.5%** | **CI 97.5%** |
| --- | --- | --- | --- | --- |
| **Between 31-90 days** | (Intercept) | 0.44 | 0.24 | 0.79 |
| Age (ref: 18-45) | 46-64 | 0.62* | 0.42 | 0.92 |
|  | 65-74 | 0.56* | 0.36 | 0.87 |
|  | 75+ | 0.47* | 0.28 | 0.79 |
| Gender (ref: male) | Female | 0.89 | 0.69 | 1.15 |
| Neighbourhood SES (ref: high) | Low | 0.9 | 0.64 | 1.25 |
|  | Medium-low | 0.52* | 0.36 | 0.75 |
|  | Medium-high | 0.69* | 0.48 | 1 |
| Deductibles depleted (ref: no) | Yes | 1.14 | 0.84 | 1.54 |
| Quarter of referral (ref: Q1) | Q2 (April – June) | 1.75* | 1.22 | 2.49 |
|  | Q3 (July – September | 1.37 | 0.95 | 1.98 |
|  | Q4 (October – December) | 1.92* | 1.39 | 2.66 |
| Comorbidities (ref: no) | Mood or anxiety | 0.91 | 0.66 | 1.26 |
|  | Other psychological complaints | 0.55 | 0.18 | 1.62 |
|  | Diabetes type II | 0.88 | 0.64 | 1.21 |
|  | Migraine | 0.52 | 0.25 | 1.08 |
|  | Cardiac arrhythmia | 2.01* | 1.28 | 3.14 |
|  | COPD | 0.76 | 0.45 | 1.3 |
|  | Heart valve disorder | 1.21 | 0.56 | 2.63 |
|  | Thyroid disorder | 1.12 | 0.71 | 1.77 |
|  | Gout | 0.82 | 0.49 | 1.37 |
|  | Rheumatoid arthritis | 0.77 | 0.38 | 1.55 |
|  | Cancer | 1.51* | 1.09 | 2.11 |
|  | Kidney | 1.33 | 0.65 | 2.71 |
| Distance to nearest hospital | Distance in km | 1.03 | 0.91 | 1.16 |
| Overlap 1^st^ COVID-19 wave (ref: no) | Yes | 0.94 | 0.72 | 1.23 |
| **Between 91-182 days** | (Intercept) | 0.02 | 0 | 0.1 |
| Age (ref: 18-45) | 46-64 | 0.43 | 0.15 | 1.27 |
|  | 65-74 | 0.31 | 0.09 | 1.06 |
|  | 75+ | 0.43 | 0.12 | 1.58 |
| Gender (ref: male) | Female | 0.75 | 0.37 | 1.53 |
| Neighbourhood SES (ref: high) | Low | 0.92 | 0.37 | 2.29 |
|  | Medium-low | 0.53 | 0.19 | 1.49 |
|  | Medium-high | 0.54 | 0.19 | 1.55 |
| Deductibles depleted (ref: no) | Yes | 2.31 | 0.78 | 6.84 |
| Quarter of referral (ref: Q1) | Q2 (April – June) | 2.38 | 0.83 | 6.83 |
|  | Q3 (July – September | 2.7* | 1 | 7.34 |
|  | Q4 (October – December) | 1.97 | 0.71 | 5.44 |
| Comorbidities (ref: no) | Mood or anxiety | 0.68 | 0.26 | 1.83 |
|  | Other psychological complaints | 1.42 | 0.17 | 11.56 |
|  | Diabetes type II | 0.62 | 0.24 | 1.55 |
|  | Migraine | 0.58 | 0.07 | 4.54 |
|  | Cardiac arrhythmia | 2.02 | 0.71 | 5.76 |
|  | COPD | 1.3 | 0.38 | 4.49 |
|  | Heart valve disorder | 5.68* | 1.76 | 18.34 |
|  | Thyroid disorder | 2.27 | 0.82 | 6.28 |
|  | Gout | 2.51 | 0.94 | 6.7 |
|  | Rheumatoid arthritis | 0.57 | 0.07 | 4.48 |
|  | Cancer | 1.97 | 0.87 | 4.51 |
|  | Kidney | 1.09 | 0.14 | 8.63 |
| Distance to nearest hospital | Distance in km | 0.97 | 0.69 | 1.38 |
| Overlap 1^st^ COVID-19 wave (ref: no) | Yes | 0.91 | 0.43 | 1.92 |
| **Between 183-366 days** | (Intercept) | 0.1 | 0.02 | 0.48 |
| Age (ref: 18-45) | 46-64 | 0.38* | 0.12 | 1.15 |
|  | 65-74 | 0.45 | 0.14 | 1.47 |
|  | 75+ | 0.62 | 0.17 | 2.29 |
| Gender (ref: male) | Female | 0.96 | 0.47 | 1.96 |
| Neighbourhood SES (ref: high) | Low | 0.58 | 0.23 | 1.45 |
|  | Medium-low | 0.53 | 0.2 | 1.4 |
|  | Medium-high | 0.53 | 0.2 | 1.4 |
| Deductibles depleted (ref: no) | Yes | 1.51 | 0.55 | 4.11 |
| Quarter of referral (ref: Q1) | Q2 (April – June) | 1.09 | 0.41 | 2.9 |
|  | Q3 (July – September | 1.01 | 0.38 | 2.66 |
|  | Q4 (October – December) | 1.09 | 0.44 | 2.72 |
| Comorbidities (ref: no) | Mood or anxiety | 0.86 | 0.34 | 2.14 |
|  | Other psychological complaints | 1.39 | 0.17 | 11.39 |
|  | Diabetes type II | 0.76 | 0.32 | 1.83 |
|  | Migraine | 0.62 | 0.08 | 4.8 |
|  | Cardiac arrhythmia | 4.64* | 1.95 | 11.04 |
|  | COPD | 2.06 | 0.75 | 5.66 |
|  | Heart valve disorder | 1.26 | 0.16 | 10.13 |
|  | Thyroid disorder | 1.03 | 0.29 | 3.64 |
|  | Gout | 2.18 | 0.81 | 5.9 |
|  | Rheumatoid arthritis | 0.6 | 0.08 | 4.68 |
|  | Cancer | 1.78 | 0.78 | 4.05 |
|  | Kidney | 0.99 | 0.12 | 8.13 |
| Distance to nearest hospital | Distance in km | 0.79 | 0.52 | 1.2 |
| Overlap 1^st^ COVID-19 wave (ref: no) | Yes | 1.2 | 0.58 | 2.51 |
| **Non-compliant** | (Intercept) | 0.05 | 0.02 | 0.13 |
| Age (ref: 18-45) | 46-64 | 0.78 | 0.44 | 1.37 |
|  | 65-74 | 0.75 | 0.41 | 1.39 |
|  | 75+ | 0.98 | 0.51 | 1.9 |
| Gender (ref: male) | Female | 0.85 | 0.61 | 1.19 |
| Neighbourhood SES (ref: high) | Low | 1.01 | 0.65 | 1.57 |
|  | Medium-low | 0.78 | 0.49 | 1.24 |
|  | Medium-high | 0.62 | 0.37 | 1.03 |
| Deductibles depleted (ref: no) | Yes | 2.77* | 1.65 | 4.65 |
| Quarter of referral (ref: Q1) | Q2 (April – June) | 1.47 | 0.95 | 2.28 |
|  | Q3 (July – September | 1.29 | 0.83 | 2.01 |
|  | Q4 (October – December) | 1.07 | 0.7 | 1.64 |
| Comorbidities (ref: no) | Mood or anxiety | 0.83 | 0.54 | 1.26 |
|  | Other psychological complaints | 0.57 | 0.13 | 2.49 |
|  | Diabetes type II | 0.89 | 0.6 | 1.33 |
|  | Migraine | 1.11 | 0.54 | 2.29 |
|  | Cardiac arrhythmia | 0.4 | 0.16 | 1.01 |
|  | COPD | 1.23 | 0.71 | 2.14 |
|  | Heart valve disorder | 0.84 | 0.29 | 2.47 |
|  | Thyroid disorder | 1.83* | 1.12 | 2.98 |
|  | Gout | 0.75 | 0.38 | 1.47 |
|  | Rheumatoid arthritis | 0.58 | 0.22 | 1.5 |
|  | Cancer | 1.62* | 1.1 | 2.4 |
|  | Kidney | 1.23 | 0.52 | 2.88 |
| Distance to nearest hospital | Distance in km | 0.9 | 0.76 | 1.06 |
| Overlap 1^st^ COVID-19 wave (ref: no) | Yes | 0.79 | 0.55 | 1.13 |
